# Scale and Pustule on Dermoscopy of Rosacea: A Diagnostic Clue for Demodex Species

**DOI:** 10.5826/dpc.1101a139

**Published:** 2021-01-29

**Authors:** Gamze Serarslan, Özlem Makbule Kaya, Emre Dirican

**Affiliations:** 1 Department of Dermatology, Mustafa Kemal University, Hatay, Turkey; 2 Department of Parasitology, Mustafa Kemal University Hatay, Turkey; 3 Department of Biostatistics, Mustafa Kemal University Hatay, Turkey

**Keywords:** *Demodex folliculorum*, *Demodex brevis*, rosacea, dermoscopy

## Abstract

**Background:**

*Demodex* mites are highly found in the skin of patients with rosacea. The diagnosis of *Demodex* can be made by standardized skin surface biopsy. Dermoscopy is a tool used in the noninvasive diagnosis of various dermatological diseases.

**Objectives:**

To determine whether dermoscopic features of demodicosis are associated with the result of standardized skin surface biopsy in patients with rosacea and to compare dermoscopic features of rosacea in *Demodex*-positive and negative samples and *Demodex* type.

**Methods:**

A total of 30 patients (7 male, 23 female) were included in the study. Dermoscopic examination was performed on both the clinically most severely affected areas and adjacent healthy skin. The skin surface biopsy sample was taken from the same place from where the dermoscopic image was taken.

**Results:**

A total of 83 (lesion n = 60, non-lesion n = 23) areas were evaluated. *Demodex* was detected in 60.2% (n = 50) of the samples. Half of these samples revealed only *Demodex folliculorum*, and the remaining half revealed *D folliculorum* and *Demodex brevis.* Of the *Demodex*-positive samples, 88% had *Demodex* tails (P =0.001) and 68% *Demodex* follicular openings (P = 0.002) on dermoscopy. In *D folliculorum*+*D brevis*-positive samples, the rate of scale and pustule was higher than *D folliculorum-*positive samples (P = 0.017 and P = 0032, respectively).

**Conclusions:**

The sensitivity and specificity of *Demodex* tail are higher than *Demodex* follicular opening and scale and pustule detection with dermoscopy and may indicate the coexistence of both *D folliculorum* and *D brevis.*

## Introduction

Rosacea is a chronic inflammatory disease that affects the face, including cheeks, chin, nose, and forehead. There is no diagnostic laboratory test for rosacea. The diagnosis and classification of rosacea are based on the clinical characteristics of the patient. Although the pathogenesis of rosacea is not fully understood, genetics, immune factors, neurovascular dysregulation, microorganisms, and environmental factors are thought to play a role. There are differences in skin flora composition, such as increased commensal organisms of skin in rosacea patients. *Demodex* species (*D folliculorum* and *D brevis*) are known commensals of facial skin. *D folliculorum* is mostly located in the hair follicle, and *D brevis* is frequently found in sebaceous and Meiboman glands [1]. *D folliculorum* is the largest member of its genus and can reach a length of 0.3–0.4 mm. *D brevis* is shorter and is 0.2–0.3mm long. The opisthosomal tip of *D folliculorum* is round, and in *D brevis* is pointed. In addition, *D folliculorum* has spurs on the legs, but *D brevis* does not. The mouthparts of *D folliculorum* are more developed than those of *D brevis* [2–4]. The number of *Demodex* mites is higher on the skin in patients with rosacea [1]. The diagnosis of *Demodex* can be made by a method called standardized skin surface biopsy (SSSB), by which it is possible to collect the superficial part of the horny layer and the complete follicle contents [5].

Dermoscopy is a tool used in the noninvasive diagnosis of various dermatological diseases such as scalp and hair diseases, nail and nail fold anomalies, and cutaneous infections (infestations and inflammatory dermatoses) [6]. *Demodex* tails (DT) and *Demodex* follicular openings (DFO) have been reported to be demodicosis-specific dermoscopic features [7].

In this study, we aimed to determine whether dermoscopic features specific to demodicosis are associated with the results of SSSB obtained from the same localization in patients with rosacea. We aimed to compare dermoscopic features of rosacea in *Demodex*-positive and negative samples and *Demodex* type.

## Methods

### Patients

This prospective study was conducted in a tertiary hospital. It was accepted by the local ethics committee. A total of 30 patients (7 male, 23 female), who were seen in the dermatology outpatient clinic and were diagnosed with rosacea, were included in the study. The diagnosis of rosacea was made according to National Rosacea Society criteria [8]. Individuals who had received any topical or systemic rosacea treatment within the previous 2 months of enrollment were excluded from the study. Information such as age, gender, duration of disease, and clinical subtype of the disease, was recorded.

### Dermoscopic Evaluation

The dermoscopic evaluation was performed by the same clinician (G.S.) by using a handheld dermoscope (DermLite DL4; 3Gen, Inc., San Juan Capistrano, USA) at ×10 magnification (cross-polarized light). Images were recorded directly by the smartphones attached magnetically to the dermoscope. Dermoscopic examination was performed on both the clinically most severely affected areas and adjacent healthy skin.

#### Dermoscopic Definitions

- *Demodex* tail; a gelatinous, whitish creamy thread, 1–3 mm in length [7].
- *Demodex* follicular opening; containing round, amorphic, grayish/light brown plugs surrounded by an erythematous halo [7].
- Dermoscopic features of rosacea; vascular structures, follicular plug, white or yellowish scale, orange yellowish areas, dilated follicles, and follicular pustules [6,9].

### Standardized Skin Surface Biopsy

An SSSB sample was taken from the same place from which the dermoscopic image was taken. An area of 1 cm^2^ was marked on a microscope slide. A drop of cyanoacrylate was placed on the other side of the slide in the middle of this area. The sample was gently pressed onto the surface and removed slowly after about 30–45 seconds. A few drops of glycerin were dropped onto the biopsy specimens and covered with coverslip. The samples were examined with a light microscope (Leica DM750, Switzerland) at ×4, ×10, and ×40 magnifications by an expert parasitologist. The diagnosis of 5 or more *Demodex* mites in 1 cm^2^ was evaluated as positive. Species identification of mites was made in accordance with the relevant literature [2].

### Statistical Analysis

Data was analyzed with 95% confidence using the SPSS for Windows (version 21; IBM Corp, Armonk, New York, USA). Frequency percent was used in the expression of clinical data and mean ± standard deviation was used in continuous variables. Chi-square test and Kappa coefficient were used to analyze the relationship of categorical variables. In addition, sensitivity and specificity values, which are among the basic measures, were included to evaluate the diagnostic performance of the developed tests.

## Results

### Demographic and Clinical Data

A total of 83 lesion areas and non-lesion areas from 30 (7 male, 23 female) patients were evaluated. The mean age of the patients was 42.50 ± 12.74 years (range, 18–72 years), and the duration of the disease was 5.41 ± 6.90 years (range, 0.125–30 years). The patients had erythematotelangiectatic (ET) (n = 17) and papulopustular (PP) (n = 13) rosacea subtypes. SSSB and dermoscopy of 83 samples were evaluated. Sixty of these samples were from the lesion area [cheek (n = 38), chin (n = 11), forehead (n = 7), nose (n = 4)] and 23 were from the normal skin area. Thirty-three of the samples taken from the lesion sites were from patients with ET rosacea, and 27 were from patients with PP rosacea. Fourteen of the samples taken from normal skin areas were from patients with ET rosacea, and 9 were from patients with PP rosacea (Table 1).

### SSB Findings

**Figure 1:**
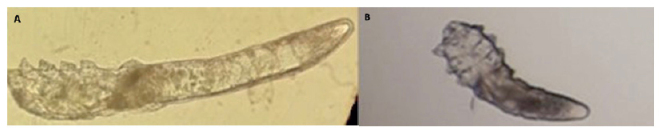
(A) *Demodex folliculorum.* (B) *Demodex brevis* (original magnification ×400).

*Demodex* was detected by SSSB in 60.2% (n = 50) of the samples. Half of these samples revealed only *D folliculorum*, and the remaining half *D folliculorum* and *D brevis* (Figure 1). All but 2 of the samples detected in *Demodex* belonged to the lesion areas.

### Dermoscopic Findings

**Figure 2:**
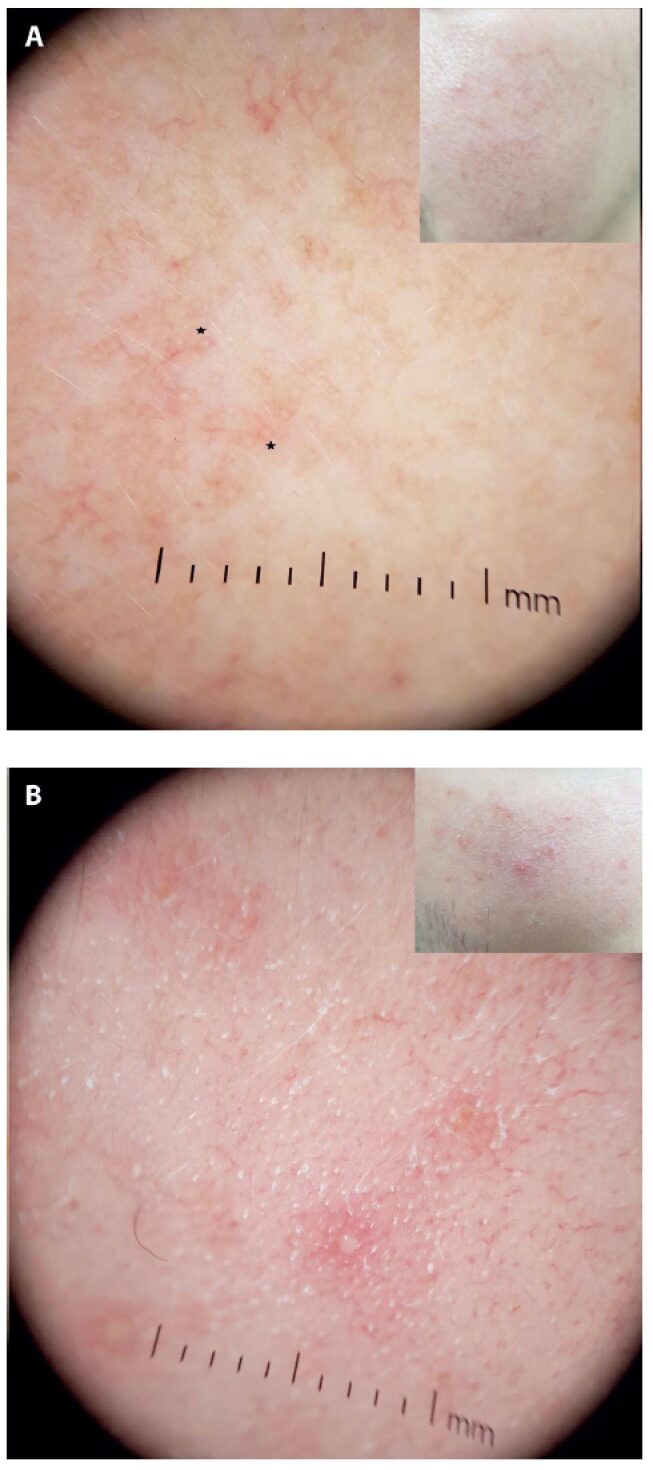
Examples of (A) *Demodex* follicular openings (stars) and (B) *Demodex* tails.

Dermoscopy revealed that DT was present in 88% (n = 44) of the samples that were positive with SSSB (P = 0.001). DFO was present in 68% (n = 34) of SSSB-positive samples (P = 0,002) on dermoscopy. Examples of DT and DFO are shown in Figure 2. Kappa value, sensitivity, and specificity of DT and DFO are shown in Table 2. There were no statistically significant differences between the *D folliculorum*-positive and *D folliculorum* + *D brevis*-positive samples in terms of DT (P = 1.00) and DFO (P = 0.363).

Dermoscopic features of the *D folliculorum*-positive lesion samples and *D folliculorum* + *D brevis*-positive lesion samples were compared. In the *D folliculorum* + *D brevis*-positive samples, the rate of scale and pustule was higher, compared to the *D folliculorum*-positive samples (P = 0.017 and P = 0.032, respectively). The details of dermoscopic features of the lesion areas according to the *Demodex* type are shown in Table 3. As reported by Lallas et al. some pustules that were not clinically noticeable could be detected in the dermoscope [8]. Dilated follicles were not included in the statistical analysis because of low rate.

**Figure 3:**
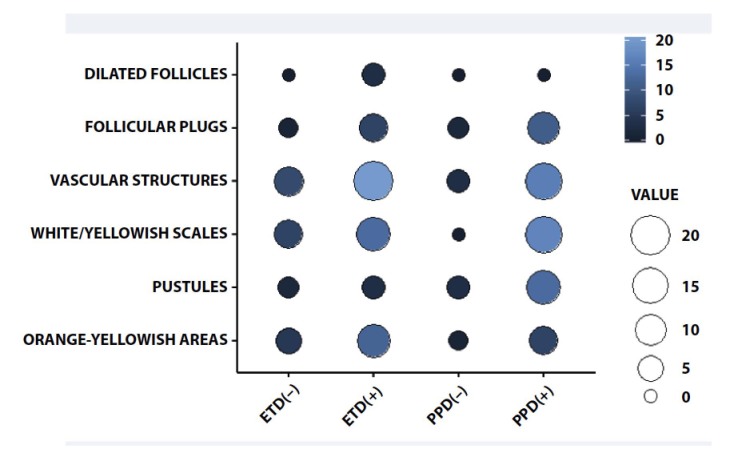
The frequency of dermoscopic features of the lesion samples with and without *Demodex*.

Lesion areas were analyzed according to the rosacea subtype. The most common dermoscopic features in both ET rosacea and PP rosacea were vascular structure (59.6% and 40.4%, respectively) and scale (54.1% and 45.9%, respectively). The dermoscopic features of *Demodex*-positive and negative lesion samples were compared and the results were as follows: follicular plugging (87.5%), vascular structures (71.4%), and orange-yellow areas (70.6%) were common in *Demodex*-positive samples of ET rosacea. Although dilated follicles were present in 100% of these samples, the number of this dermoscopic feature was low (n=3). Scale (100%), orange-yellow areas (87.5%), and follicular plugging (84.6%) were frequently detected dermoscopic findings in the *Demodex*-positive samples of PP rosacea (Figure 3).

## Conclusions

We found that the dermoscopic findings of DT and DFO were statistically significant in terms of the presence of *Demodex* (P = 0.001 and P = 0.002, respectively). The sensitivity and specificity of DT and DFO were 0.88/0.73 and 0.68/0.67, respectively. There are a few studies on the relationship between DT and DFO findings and *Demodex* in dermoscopy. Segal et al. were the first to describe these 2dermoscopic features associated with *Demodex*. The authors reported that the dermoscopy findings showed excellent agreement with the microscopic findings [7]. It was also reported that the tails are less abundant in the inflammatory forms of demodicosis including rosacea-like demodicosis [7]. In a study conducted in patients with demodicosis including rosacea, it was reported that DT was the only specific and sensitive criterion in the diagnosis [11]. In another study, the sensitivity and specificity of the DT were reported as 66.7% and 100%; the sensitivity and specificity of the DFO were reported as 54.8% and 97%, respectively, in patients with *Demodex-*associated folliculitis [12]. In the studies mentioned above, some of the results are not compatible with each other—including our study. The reason for this may be the difference in the patient groups studied.

The superficial layer of the horn layer and the pilosebaceous follicle content can be collected by SSSB. However, not all biotopes of *D folliculorum* can be collected with SSSB, and it can cause false-negative results. Forton et al. offered to perform a second SSSB at the same place [13]. Although DT was detected on dermoscopy, SSSB was negative in 9 of 83 samples in our study. This may be because no sample was taken from the same place more than once. In addition, the number of samples taken from the control area was less than the number of samples taken from the lesion area. This is the limitation of our study.

In the *D folliculorum* + *D brevis*-positive samples, the rate of scale and pustule was higher compared to the *D folliculorum*-positive samples. Karadağ Köse et al. reported similarly that demodicosis might be suspected in the presence of epidermal scale [11].

The results of our study can generally be evaluated as follows: (1) Although DT and DFO indicate *Demodex* on dermoscopy, the sensitivity and specificity of DT are higher than the DFO. (2) Scale and pustule detection on dermoscopy may indicate the coexistence of both *D folliculorum* and *D brevis.*

## Figures and Tables

**Table 1:** Clinical and Demographic Characteristics of the Patients and Lesions

Characteristics	n (%)
**Sex**	
Female	23 (76.6)
Male	7 (23.4)
**Age (years); mean ± SD**	42.50 ± 12.74
**Duration of disease(years); mean ± SD**	5.41 ± 6.90
**Rosacea subytpe**	
Erythematotelangiectatic	17 (56.6)
Papulopustular	13 (43.4)
**Total number of samples**	
Lesions	60 (72.2)
Controls	23 (27.8)
**Location of lesions**	
Cheek	38 (63.3)
Chin	11 (18.3)
Forehead	7 (11.6)
Nose	4 (6.8)

**Table 2:** P Value, Kappa Value, Sensitivity and Specifity of *Demodex* Tail and *Demodex* Follicular Opening

		*Demodex (−)*	*Demodex (+)*	P value	Kappa	Sensitivity	Specifity
***Demodex*** ** tail**	**(−)**	24 (72.7)	6 (12.0)	0.001*	0.617	0.88	0.73
	**(+)**	9 (27.3)	44 (88.0)				
**Dilated follicular opening**	**(−)**	22 (66.7)	16 (32.0)	0.002*	0.338	0.68	0.67
	**(+)**	11 (33.3)	34 (68.0)				

**Table 3:** Dermoscopic Features of Lesion Areas According to the *Demodex* Type

		Demodex type		Total	P value
		*D folliculorum* n (%)	*D folliculorum + D brevis* n (%)		
Scale	(−)	13 (54.2)	5 (20.8)	18	0.017
	(+)	11 (45.8)	19 (79.2)	30	
Pustule	(−)	20 (83.3)	12 (50.0)	32	0.032
	(+)	4 (16.7)	12 (50.0)	16	
Follicular plug	(−)	17 (70.8)	13 (54.2)	30	0.371
	(+)	7 (29.2)	11 (45.8)	18	
Vascular structures	(−)	4 (16.7)	8 (33.3)	12	0.317
	(+)	20 (83.3)	16 (66.7)	36	
Orangeyellowish area	(−)	13 (54.2)	16 (66.7)	29	0.555
	(+)	11 (45.8)	8 (33.3)	19	
